# Robust Tracker: Integrating CPM-YOLO and BOTSORT for Cross-Modal Vessel Tracking

**DOI:** 10.3390/s26030983

**Published:** 2026-02-03

**Authors:** Feng Lv, Ying Zhang

**Affiliations:** College of Information Engineering, Shanghai Maritime University, Shanghai 201306, China

**Keywords:** small-object detection, BOTSORT, ReID, multi-object tracking

## Abstract

This paper presents a high-accuracy and robust multi-object tracking method for maritime vessel detection and tracking in complex marine environments, characterized by dense targets, large-scale variations, and frequent occlusions. The proposed approach adopts an enhanced YOLOv8-based detector with lightweight feature enhancement and attention mechanisms to improve its capability in detecting small-scale vessels and complex scenes. Furthermore, a tracking framework combining BOTSORT with an OSNet-based re-identification (ReID) model is employed to achieve stable and reliable vessel association. Experimental results on the Near-Infrared On-Shore (NIR) dataset demonstrate that the proposed method improves Precision, Recall, mAP@0.5, and mAP@0.5:0.95 by approximately 4.0%, 5.0%, 5.1%, and 5.4%, respectively, compared with the baseline YOLOv8, while reducing parameter count and model size by 11.6% and 6.5%. On the Visible On-Shore (VIS) dataset, the proposed method outperforms state-of-the-art approaches in detection accuracy and robustness, further validating its effectiveness and cross-modal generalization capability. In multi-object tracking tasks, the proposed CPM-YOLO and BOTSORT framework demonstrates clear advantages in trajectory continuity, occlusion handling, and identity preservation compared with mainstream tracking algorithms. On the NIR dataset, the proposed method achieves a competitive inference speed of 188 FPS, while running at 187 FPS on the VIS dataset, demonstrating that the accuracy improvements are achieved without sacrificing real-time performance. Overall, the proposed method achieves a favorable balance between detection accuracy, tracking robustness, and model efficiency, making it well-suited for practical maritime applications.

## 1. Introduction

With the rapid development of global maritime traffic and intelligent vessel applications, multi-target detection and tracking of ships has become one of the core technologies in fields such as intelligent ocean monitoring, port security, and channel management [1]. Multi-object tracking (MOT) not only requires accurate detection of multiple targets in a scene, but also demands continuous association of the same target’s trajectory over time to support subsequent dynamic behavior analysis and risk warning [2]. However, due to the impact of complex marine environments, such as densely distributed targets, large-scale variation, frequent occlusions, and background interference, traditional detection and tracking methods face significant challenges in practical applications [3,4].

In recent years, deep learning-driven end-to-end detection and tracking frameworks have made significant progress, with methods represented by the YOLO series object detection algorithms and detection-based trackers gaining widespread application in the MOT field [5,6,7]. These methods combine efficient detectors with robust data association mechanisms to achieve real-time detection and tracking of multiple targets in complex dynamic scenes. However, existing methods often face significant challenges, such as inaccurate target association, decreased identity consistency, and insufficient tracking robustness when dealing with complex scenarios involving a high proportion of small targets, similar target appearances, and dense occlusion.

To address these challenges, the development of multi-object tracking methods with high detection accuracy, efficient feature representation, and strong robustness has attracted increasing research attention. This study proposes an end-to-end method tailored for complex maritime scenes, integrating efficient detection with robust tracking. By incorporating a lightweight feature extraction module, multi-scale feature fusion mechanism, and advanced target association algorithms, the proposed method significantly enhances the overall performance of the maritime multi-object tracking system in terms of accuracy, efficiency, and engineering applicability.

This paper proposes a multi-object ship detection and tracking method tailored for complex maritime multimodal scenarios, including both Visible On-Shore (VIS) and Near-Infrared On-Shore (NIR) imaging. All core modules are designed to be adaptable to different imaging modalities, and systematic evaluations are conducted on both NIR and VIS datasets. By integrating an optimized YOLOv8 detector and the BOTSORT tracker, the proposed framework achieves high-precision, real-time, and robust multi-ship tracking under challenging conditions. The main contributions are as follows:

1. To address the low target–background contrast and insufficient discriminative features in infrared imagery, a novel lightweight feature extraction module, termed C2fCPCAGhost, is proposed for infrared-aware feature modeling in complex maritime scenes. Built upon the C2f structure, this module integrates GhostConv with Channel Prior Convolution Attention (CPCA), enhancing key feature responses while significantly reducing model complexity, and demonstrating strong adaptability to both visible and infrared modalities, particularly for low-contrast infrared targets.

2. Considering dense small-object distributions and long-range infrared observation in complex maritime scenes, a dedicated P2 small-object detection branch is designed and selectively enhanced with Multi-Head Self-Attention (MHSA) to explicitly compensate for the limited global modeling capability of convolutional features under infrared long-range observation. By incorporating high-resolution features and explicitly modeling global dependencies, this design effectively improves the detection and discrimination of multi-scale, small, and densely distributed targets, yielding enhanced accuracy and stability in both visible and infrared scenarios.

3. To mitigate appearance ambiguity and identity confusion in infrared-based multi-object tracking, a tracking framework integrating an Omni-Scale Network (OSNet)-based re-identification (ReID) model with the BOTSORT tracker is constructed. Through the collaborative use of appearance and motion cues, this framework significantly reduces identity (ID) switches under infrared weak-appearance and occlusion conditions, thereby improving the continuity and robustness of maritime multi-object tracking.

## 2. Related Works

MOT is a fundamental research topic in intelligent vision systems and has received increasing attention in applications such as autonomous driving, video surveillance, and intelligent maritime perception. The goal of MOT is to simultaneously perform accurate object detection and maintain consistent identities of multiple targets across consecutive frames, which directly affects system robustness and practical reliability in complex environments [8].

Early MOT methods mainly relied on motion modeling and data association strategies. Simple Online and Realtime Tracking (SORT) [9] employed Kalman filtering and the Hungarian algorithm to achieve efficient online tracking with low computational cost. However, such motion-only approaches are highly sensitive to occlusion, dense object distributions, and appearance similarity, often resulting in frequent identity switches and mismatches. To address this limitation, DeepSORT [10] introduced deep appearance features into the association process, significantly improving tracking robustness under partial occlusion and appearance ambiguity. Building upon this paradigm, more recent methods such as OCSORT, DeepOCSORT, and BOTSORT further enhanced association stability by incorporating observation-centric modeling, adaptive re-identification features, and refined matching strategies, achieving improved identity consistency while maintaining real-time performance [6,11,12]. These approaches currently represent state-of-the-art (SOTA) solutions within the detection-based MOT framework.

Meanwhile, the rapid development of deep neural networks has driven continuous advances in object detection. Single-stage detectors from the YOLO family have been widely adopted due to their end-to-end architecture and high inference efficiency, demonstrating strong performance across diverse application scenarios [13,14,15,16,17]. As a result, YOLO-based detectors have become popular front-end components in many MOT systems [18,19,20]. To further improve detection performance in complex environments, researchers have introduced feature pyramid structures, multi-branch feature fusion, and attention mechanisms to enhance multi-scale representation and robustness to appearance variations [21,22]. These improvements have proven particularly effective for small-object detection and dynamic scene adaptation [23,24,25], providing more reliable observations for downstream tracking and trajectory modeling. Small-object detection has also been actively studied in engineering and safety-related domains, where targets are often small and densely distributed and images are captured under challenging viewpoints (e.g., UAV imagery and images captured by surveillance cameras and body-worn cameras). Wang [26] systematically evaluated traditional augmentation strategies for UAV-based rebar counting with Faster R-CNN and YOLOv10-based transformer architectures across multiple backbones, showing that augmentation effectiveness is architecture- and metric-dependent and can improve average precision and counting accuracy. In a related study, a YOLOv10-based framework was benchmarked with a convolutional neural network and transformer-based backbones for safety-helmet monitoring, reporting strong detection performance in real-world monitoring settings [27]. These cross-domain efforts highlight the importance of robust multi-scale representations and practical deployment considerations, which are also central to the maritime detection-and-tracking scenario studied in this paper. Recent studies have further validated the effectiveness of YOLOv8 as a strong baseline for detection in challenging real-world scenarios. For example, Giri et al. [28] proposed SO-YOLOv8 to enhance small-object detection beyond the COCO benchmark, demonstrating improved robustness under scale variation and low-resolution conditions. Similarly, Chen et al. [29] developed an enhanced YOLOv8 framework for road surface damage detection, achieving superior accuracy and stability in complex and cluttered environments. These works indicate that YOLOv8 provides a flexible and reliable foundation for task-specific architectural enhancements, which motivates its adoption in this study.

In addition to modular detection-and-tracking pipelines, joint detection and tracking frameworks have emerged as an active research direction [30,31]. Representative methods such as FairMOT and CenterTrack adopt end-to-end designs that jointly optimize detection, feature learning, and data association, thereby reducing mismatches and improving performance in crowded and occluded scenes [32,33]. However, such unified frameworks often require substantial computational resources and may suffer from limited real-time performance and generalization capability, especially in complex maritime environments and multimodal imaging conditions.

Despite the progress achieved by existing SOTA methods, multi-object detection and tracking in maritime scenarios remain challenging. Issues such as large-scale variations, high appearance similarity among vessels, frequent occlusions, and low target–background contrast under infrared imaging conditions make it difficult to simultaneously achieve high detection accuracy, stable identity association, and computational efficiency. Therefore, developing a lightweight, accurate, and robust detection–tracking framework tailored for complex maritime environments remains an open and important research problem, which motivates the work presented in this paper.

## 3. Robust Tracker

### 3.1. CPM-YOLO Model Architecture

**Figure 1:**
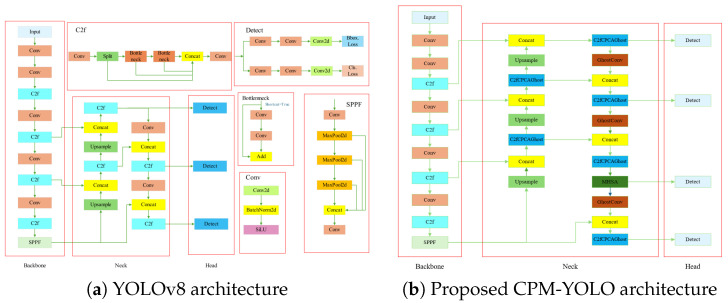
Architectural comparison between the original YOLOv8 and the proposed CPM-YOLO. The CPM-YOLO architecture preserves the overall backbone and detection paradigm of YOLOv8, while introducing three key modifications: (1) the C2fCPCAGhost module in the neck, (2) a dedicated small-object detection branch (P2), and (3) an MHSA mechanism to enhance feature representation and robustness in complex maritime scenarios.

To meet the joint requirements of detection accuracy, robustness, and real-time performance in maritime multi-object tracking under complex multimodal scenarios, this paper proposes a lightweight multi-branch detection network, termed CPM-YOLO, built upon the YOLOv8 baseline. The proposed model preserves the efficient and stable feature extraction capability of the YOLOv8 backbone, while introducing targeted architectural modifications that distinguish CPM-YOLO from the original YOLOv8, mainly in the neck and detection heads. Specifically, the original C2f modules in the neck are systematically replaced with the proposed C2fCPCAGhost modules, which integrate the efficient feature generation of GhostConv with CPCA to enhance feature discriminability while significantly reducing model complexity, particularly improving responses to low-contrast targets in infrared imagery. In addition, a P2 small-object detection branch is introduced to incorporate high-resolution fine-grained features, effectively improving the detection of small-scale and densely distributed vessels. Furthermore, MHSA is additionally embedded into selected detection heads in CPM-YOLO to explicitly model global feature dependencies, enhancing detection stability under frequent occlusion and complex backgrounds. Through these collaborative designs, CPM-YOLO achieves a favorable balance between lightweight deployment and real-time performance, while demonstrating strong adaptability to both visible and near-infrared imaging scenarios. A side-by-side architectural comparison between YOLOv8 and CPM-YOLO is provided in Figure 1.

#### 3.1.1. Design of the C2fCPCAGhost Module

In multi-object detection and tracking tasks, the feature extraction module must achieve an effective balance among representation capacity, computational efficiency, and robustness. The C2f module in YOLOv8, benefiting from its compact structure and multi-branch feature aggregation mechanism, demonstrates high efficiency in general object detection. However, in complex maritime scenarios, vessel targets are often densely distributed, dominated by small-scale objects, heavily affected by background interference, and exhibit low target–background contrast under infrared imaging conditions. These characteristics limit the capability of the original C2f module in dynamic feature selection, cross-channel discriminative modeling, and fine-grained target representation, making it difficult to simultaneously satisfy the requirements of detection accuracy and tracking robustness.

**Figure 2:**
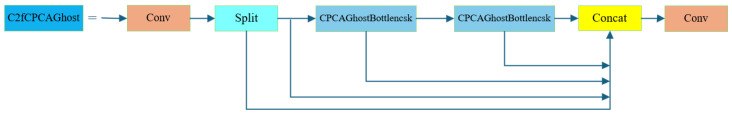
C2fCPCAGhost module architecture. This module integrates GhostBottleneck and CPCA to enhance feature representation and achieve efficient, lightweight multi-scale fusion.

To address these challenges, this paper proposes a lightweight feature extraction module, termed C2fCPCAGhost, specifically designed for complex maritime environments by jointly optimizing feature generation efficiency and channel-wise discriminative modeling. Building upon the split–aggregation structure of C2f, the proposed module introduces CPCAGhostBottleneck as the core computational unit, which integrates the efficient feature generation of GhostBottleneck with CPCA. This design effectively suppresses feature redundancy and adaptively enhances key channel responses, thereby improving feature discriminability and stability while significantly reducing parameter size and computational overhead. The overall structure of the module is illustrated in Figure 2.

Within the C2fCPCAGhost module, the CPCAGhostBottleneck serves as the core unit that jointly performs efficient feature generation and dynamic feature selection. Unlike conventional bottleneck structures that rely solely on fixed convolutional kernels, this unit is built upon a lightweight GhostBottleneck backbone and further enhanced by integrating the CPCA. By adaptively modeling the relative importance of different channel features, CPCAGhostBottleneck effectively emphasizes target-relevant discriminative information while suppressing redundant and noisy responses, thereby improving robustness under complex backgrounds and low-contrast imaging conditions.

**Figure 3:**
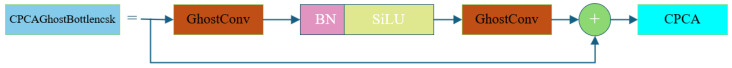
CPCAGhostBottleneck module architecture. The module employs GhostConv layers with batch normalization and SiLU activation, followed by residual connection and CPCA to enhance feature representation with low computational overhead.

Specifically, CPCAGhostBottleneck first employs two successive GhostConv operations to construct efficient pathways for generating primary and inexpensive feature maps, enabling rich feature representations with low computational cost. A residual connection is then introduced to preserve the original input information and alleviate feature degradation during deep propagation. Finally, CPCA is applied to the aggregated features to perform channel-wise reweighting, further enhancing critical feature responses and improving overall discriminability and robustness. The detailed architecture of CPCAGhostBottleneck is illustrated in Figure 3.

In lightweight deep neural network design, GhostConv is an efficient convolution operator whose core idea is to generate discriminative primary feature maps using only a small number of standard convolutional kernels, while the remaining redundant features are derived from these primary features through computationally inexpensive linear transformations, such as depthwise separable or pointwise convolutions. This strategy significantly reduces parameter size and computational complexity while preserving feature diversity and representational capacity.

**Figure 4:**
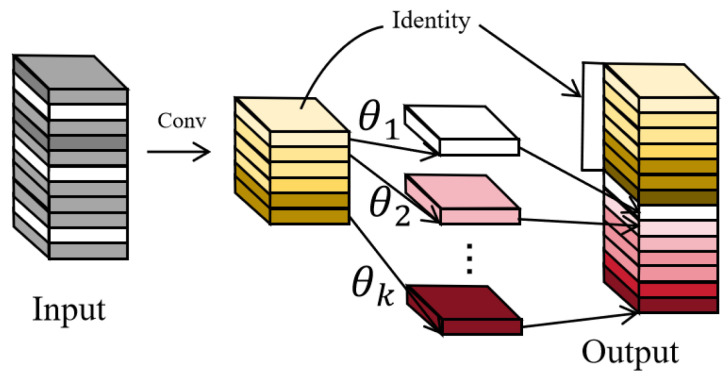
Illustration of the Ghost module. The GhostConv adopts a two-step feature generation strategy, where intrinsic feature maps are first produced by standard convolution, and additional ghost features are then generated through inexpensive linear operations to complete the feature representation efficiently.

In the proposed CPCAGhostBottleneck structure, GhostConv is adopted as the fundamental feature generation unit and is applied twice in a cascaded manner along both the main and residual paths, enabling efficient feature extraction and effective information preservation. On the one hand, this design fully exploits the high efficiency of GhostConv to reduce the overall computational burden of the model; on the other hand, it provides compact and information-rich feature representations for subsequent attention modeling. Compared with conventional convolution operations, GhostConv substantially lowers resource consumption while maintaining or even enhancing feature discriminability, thereby satisfying the dual requirements of efficiency and accuracy in multi-object detection and tracking tasks under complex maritime scenarios. The architectural illustration of GhostConv is shown in Figure 4.

The output feature of a conventional convolutional layer for input $X\in R^{c\times h\times w}$ can be expressed as

(1) Y=X∗f+b

GhostConv obtains the main feature map $Y^{\prime }$ with fewer convolution kernels, while the remaining features are generated through inexpensive linear operations:

(2) $$Y^{\prime }=X*f^{\prime }$$

Although GhostConv enables efficient feature generation with extremely low parameter and computational costs, it primarily focuses on local spatial modeling and exhibits limitations in channel-wise discriminability and global contextual dependency modeling, especially in complex scenes. In maritime environments characterized by significant scale variations, dense target distributions, and strong background interference, relying solely on local convolutional operations often leads to insufficient inter-channel interaction, resulting in feature redundancy and constrained representation capacity, which adversely affects the stability and robustness of downstream detection and tracking tasks.

**Figure 5:**
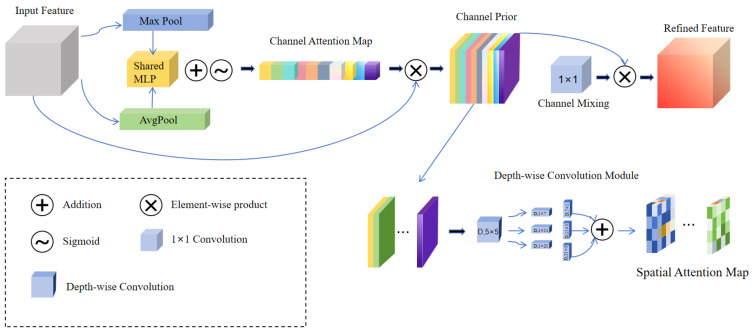
Architecture of the channel prior convolution attention module. The module enhances feature representation and global modeling capability through the fusion of channel attention and spatial attention mechanisms.

To address these limitations, CPCA is introduced into the CPCAGhostBottleneck to enhance dynamic feature modeling and contextual awareness, as illustrated in Figure 5. CPCA integrates channel prior modeling with depthwise separable convolution operations to adaptively estimate the importance of different channels. By suppressing redundant responses while emphasizing discriminative features, CPCA significantly improves feature separability and global semantic modeling. This design effectively compensates for the deficiencies of GhostConv in channel modeling and contextual perception, enabling a synergistic integration of lightweight feature generation and highly discriminative feature representation.

In summary, the proposed C2fCPCAGhost module integrates the CPCAGhostBottleneck, effectively combining the efficient feature generation capability of GhostConv with the CPCA mechanism. This synergistic design enables deep modeling of discriminative features across multiple scales in complex maritime scenarios. While preserving model lightweightness and real-time performance, the proposed module significantly enhances feature discriminability and robustness, providing a solid and reliable feature foundation for subsequent high-precision multi-object detection and stable tracking.

#### 3.1.2. Small-Object Detection Layer Design

In real-world maritime scenarios characterized by complex backgrounds and significant scale variations, small objects often occupy only a limited number of pixels, exhibit sparse texture information, and are easily disturbed by background clutter. As a result, discriminative cues of small targets are prone to attenuation or loss during deep feature extraction and downsampling processes, leading to frequent missed detections and false alarms in mainstream detection networks. This issue becomes particularly pronounced under long-range observation and infrared imaging conditions.

To address these challenges, a dedicated P2 detection branch targeting small-scale objects is introduced into the feature fusion architecture. Without altering the overall multi-scale detection framework, this branch explicitly incorporates shallow high-resolution features into the detection pathway, enabling effective exploitation of fine-grained spatial structural information. By aggregating low-level detailed features with high-level semantic representations, the P2 branch compensates for the insufficient discriminative information of small targets in deep features, facilitating cross-layer fusion and multi-scale feature propagation. Compared with conventional detection structures that rely solely on high-level features for prediction, the proposed P2 branch significantly enhances the model’s sensitivity to small-scale and densely distributed targets, allowing more stable preservation of object boundaries and local structures in complex maritime backgrounds, thereby improving detection accuracy and localization reliability.

Moreover, the P2 small-object detection branch provides more accurate and stable candidate detections for subsequent multi-object tracking modules, effectively reducing initialization errors and ID drift risks. From a system-level perspective, this design further enhances the continuity and robustness of ship multi-object tracking, establishing a reliable foundation for intelligent maritime monitoring and related engineering applications.

#### 3.1.3. Introduction of MHSA Module

In complex maritime environments, ship multi-object detection and tracking are often challenged by dense target distributions, frequent occlusions, and high inter-class visual similarity. Due to the inherently limited receptive field of convolutional operators, conventional convolutional neural networks struggle to effectively capture long-range dependencies among targets during feature modeling. This limitation frequently leads to object merging, class confusion, and degraded detection accuracy in crowded and occluded scenarios, thereby undermining the stability of subsequent tracking.

**Figure 6:**
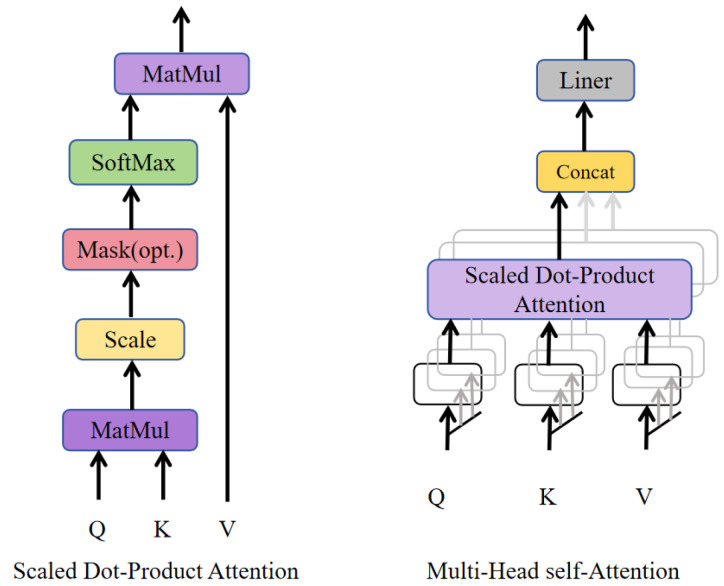
Architecture of the MHSA module. The module enables parallel attention modeling across multiple heads to capture global feature dependencies in complex scenes. The arrows indicate the direction of data flow during the attention computation process. Q, K, and V denote the query, key, and value matrices used in the self-attention mechanism.

To address these issues, an MHSA mechanism is incorporated into the multi-branch feature fusion architecture, as illustrated in Figure 6. By performing parallel attention operations across multiple heads, MHSA adaptively reweights feature responses at different spatial positions within distinct subspaces, enabling explicit modeling of global dependencies among features. This mechanism facilitates dynamic information interaction across spatial locations and significantly enhances the network’s capability to capture long-range target correlations, thereby improving feature discrimination in scenarios involving occlusion, dense distributions, and visually similar objects.

In the proposed CPM-YOLO framework, the MHSA module is deployed in selected detection output branches within the Neck to complement the limited global modeling capacity of convolutional features. Without introducing substantial computational overhead, this design strengthens the detection head’s utilization of global contextual information, leading to improved target separation and detection stability in complex environments. Moreover, the resulting more accurate and consistent detections provide high-quality observations for downstream multi-object tracking, effectively reducing association ambiguities under occlusion conditions.

#### 3.1.4. Integration of the ReID Model

In complex maritime multi-ship tracking scenarios, factors such as high inter-target appearance similarity, constrained imaging conditions, and frequent occlusions make tracking methods that rely solely on motion cues or short-term appearance features prone to identity ambiguity and ID switches. This issue is particularly pronounced under infrared imaging conditions, where limited texture information and low appearance discriminability impose stricter requirements on maintaining cross-frame identity consistency. To address this challenge, a ReID model is introduced on top of the detection results to enhance target discrimination and identity stability in complex environments.

**Figure 7:**
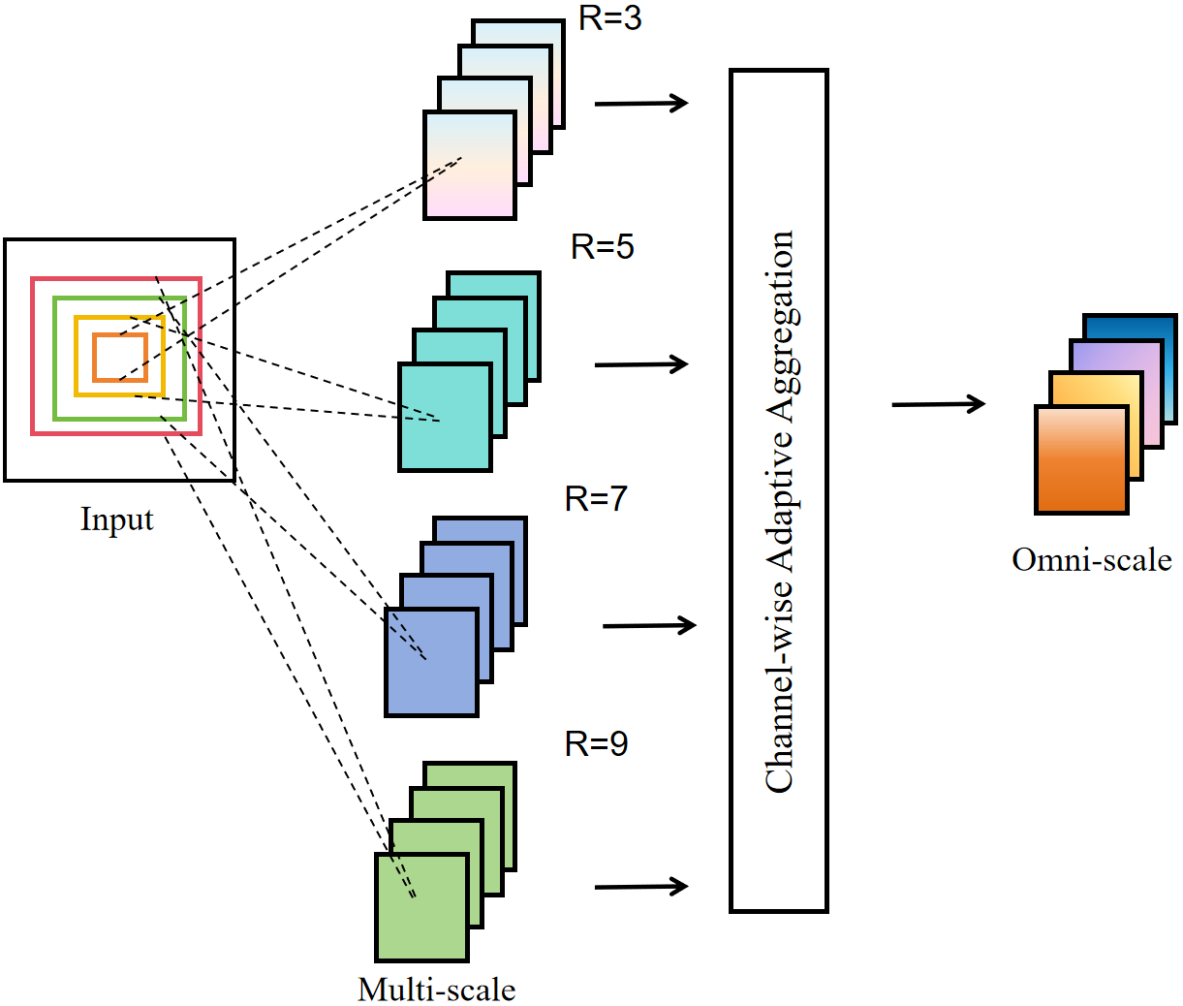
Architecture of the OSNet-based ReID feature extraction network. The network extracts multi-scale features using convolutional kernels with different receptive fields (R = 3, 5, 7, 9) and adaptively aggregates them to form discriminative omni-scale feature representations.

In this work, OSNet is adopted as the ReID feature extraction network and integrated into the BOTSORT tracking framework. OSNet employs parallel multi-scale convolutional streams to model feature responses under different receptive fields, and leverages channel-adaptive weighting to achieve dynamic fusion of multi-scale features [34]. As illustrated in Figure 7, this architecture captures both fine-grained local details and global semantic information, forming highly discriminative omni-scale representations, which are particularly effective for distinguishing visually similar targets.

Within the proposed framework, appearance features extracted by OSNet serve as an important complementary cue for target association, jointly participating in the cross-frame matching process together with motion prediction and IoU constraints in BOTSORT. Without significantly increasing system complexity, this design substantially enhances identity consistency verification, especially under occlusion recovery, dense ship traffic, and infrared weak-appearance conditions, effectively reducing ID switches and false associations. Consequently, the ReID module works synergistically with the front-end CPM-YOLO detector, providing a more stable and robust overall solution for maritime multi-object tracking.

## 4. Experimental Validation and Results Analysis

### 4.1. Experimental Setup and Parameter Configuration

All experiments in this study were conducted on a high-performance server with the following hardware configuration: NVIDIA RTX 3090 GPU (25.4 GB VRAM), 16-core AMD EPYC 7542 processor, and 60.1 GB of RAM. The software environment includes Ubuntu 22.04, CUDA version 13.0, Python version 3.11.8, and PyTorch 2.2.2. The AdamW optimizer was used for training with a batch size of 80, up to 150 epochs, and an early stopping strategy (patience = 50) to prevent overfitting.

### 4.2. Datasets

This study uses two public maritime datasets: the NIR subset and the VIS dataset from the SMD [35]. The NIR subset contains 30 near-infrared video sequences, covering various vessel categories, sizes, weather conditions, and motion patterns. Each video sequence is annotated with bounding boxes and vessel IDs on a per-frame basis. To reduce redundancy, 23 of the videos are sampled by extracting every third frame, and the data is split into training and validation sets with a 7:3 ratio. The remaining 7 videos are used as an independent test set to evaluate the performance of the tracking algorithm in novel scenes. The VIS dataset includes 40 visible-light video sequences, covering various vessel types and complex scenes. To increase diversity, every third frame is extracted in the VIS dataset as well, with 90% of the sequences split into training and validation sets (7:3 ratio), and the remaining 10% used for testing in unseen scenarios. By integrating diverse data across different spectra, the datasets provide a solid foundation for evaluating subsequent target detection and tracking algorithms.

### 4.3. Evaluation Metrics

The performance of the proposed model is evaluated using standard metrics, including Precision, Recall, and mean Average Precision (mAP). These metrics comprehensively reflect the effectiveness of detection and tracking tasks. Specifically, Precision measures the proportion of true positives among all targets detected as positive, reflecting the model’s ability to reduce false positives:

(3) $$P=\frac{TP}{TP+FP}$$

Recall measures the proportion of true positives correctly identified by the model among all actual positive samples. Its calculation formula is as follows:

(4) $$R=\frac{TP}{TP+FN}$$

Mean Average Precision is used to measure overall detection performance. Its definition is as follows:

(5) $$\mathrm{mAP}=\frac{1}{N}\sum_{i=1}^{n}AP_{i}$$

### 4.4. Experimental Results and Analysis

#### 4.4.1. Vessel Detection Model Comparison

To evaluate the proposed method’s vessel detection performance under different imaging conditions, we conducted experiments on two datasets: NIR and VIS. Comparison models include YOLOv5, YOLOv7, YOLOv8, YOLOv11, and YOLOv12. The metrics used include Precision, Recall, mAP@0.5, mAP@0.5:0.95, inference speed (FPS), model parameters, and model size. Results are shown in Table 1 (NIR) and Table 2 (VIS).

For a fair and unbiased comparison, all baseline detectors (YOLOv5, YOLOv7, YOLOv8, YOLOv11, and YOLOv12) were trained and evaluated under a unified experimental protocol, including identical optimizer settings, batch size, training epochs, input resolution (640 × 640), and Mosaic Augmentation, as detailed in Section 4.1. All detectors were initialized with their official pretrained weights.

The proposed method outperforms all comparison models on both datasets, demonstrating superior detection accuracy and compactness. On the NIR dataset, it achieves Precision, Recall, mAP@0.5, and mAP@0.5:0.95 of 95.3%, 76.1%, 82.3%, and 57.5%, respectively, outperforming YOLOv8 by 5.4% in mAP@0.5:0.95. Compared with YOLOv8, the proposed method maintains a comparable inference speed of 188 FPS on the NIR dataset, indicating that the accuracy gains are achieved without introducing additional computational overhead. On the VIS dataset, the proposed method achieves 94.7%, 76.8%, 81.4%, and 56.8%, improving mAP@0.5:0.95 by 6.2% over YOLOv8. Similarly, the proposed method runs at 187 FPS on the VIS dataset, further demonstrating that the performance improvements do not compromise real-time inference capability. This indicates that the proposed model maintains robust performance across both low-contrast NIR and complex VIS scenarios.

Additionally, the proposed method has significantly fewer parameters and a smaller size than mainstream models, making it suitable for rapid deployment in real-world applications and on low-resource devices. Combined with its competitive inference speed, the proposed method provides an effective balance between computational efficiency and detection performance. In conclusion, the proposed method achieves a strong balance between detection accuracy and model efficiency, with robust generalization and practical applicability in complex maritime environments.

#### 4.4.2. Ablation Study of Vessel Detection Models

To evaluate the contribution of each module and its applicability across different imaging modalities, we conducted ablation experiments on the NIR and VIS datasets, with results shown in Table 3 and Table 4. Using YOLOv8 as the baseline, we introduced the C2fCPCAGhost module, the P2 small-object detection branch, and the MHSA mechanism to assess their effects on accuracy, recall, multi-scale adaptability, and model complexity.

Replacing the C2f module with C2fCPCAGhost improved performance on both datasets. On the NIR dataset, mAP@0.5:0.95 increased from 52.1% to 53.6%, and parameters decreased from 3.01M to 2.56M, with a similar trend observed on the VIS dataset. This shows that C2fCPCAGhost enhances feature expression while maintaining model compactness.

**Figure 8:**
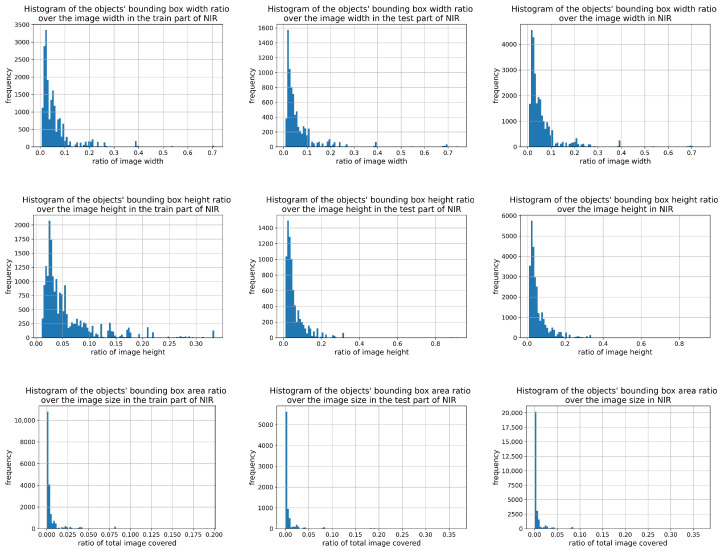
Proportional distributions of vessel target sizes in image frames of the NIR dataset. From left to right, the columns correspond to the training set, test set, and complete dataset. From top to bottom, the rows show the distributions of bounding box width ratio, height ratio, and area ratio, respectively.

**Figure 9:**
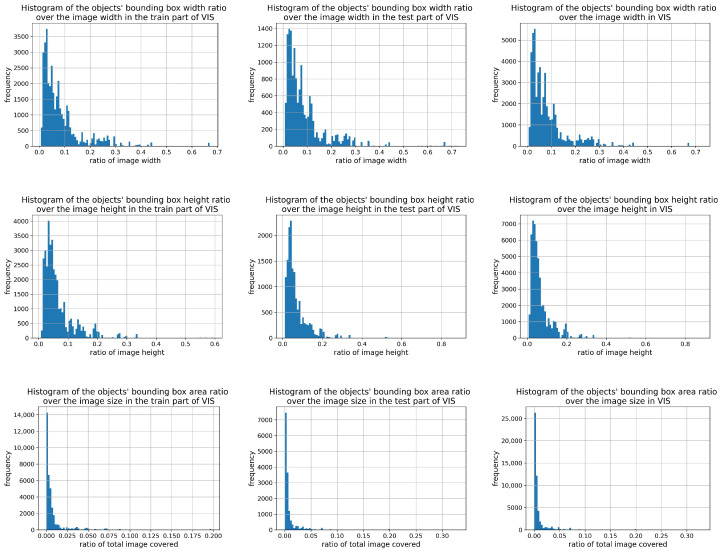
Proportional distributions of vessel target sizes in image frames of the VIS dataset. From left to right, the columns correspond to the training set, test set, and complete dataset. From top to bottom, the rows show the distributions of bounding box width ratio, height ratio, and area ratio, respectively.

A statistical analysis of target sizes in both datasets (Figure 8 and Figure 9) revealed that small targets dominate, especially in the NIR dataset. This highlights the importance of small-object detection, validated by the improvement in recall from 71.2% to 75.2% on the NIR dataset after adding the P2 branch. This also resulted in a consistent performance increase on the VIS dataset.

Finally, incorporating the MHSA mechanism optimized model performance, with mAP@0.5:0.95 rising to 57.5% on NIR and 56.8% on VIS. This demonstrates the effectiveness of global attention in modeling complex relationships and long-range dependencies.

In conclusion, the integration of lightweight feature enhancement, small-object detection, and global attention resulted in optimal performance across both datasets, validating the effectiveness and cross-modal robustness of the proposed model.

#### 4.4.3. Visual Results of Vessel Detection Performance

**Figure 10:**
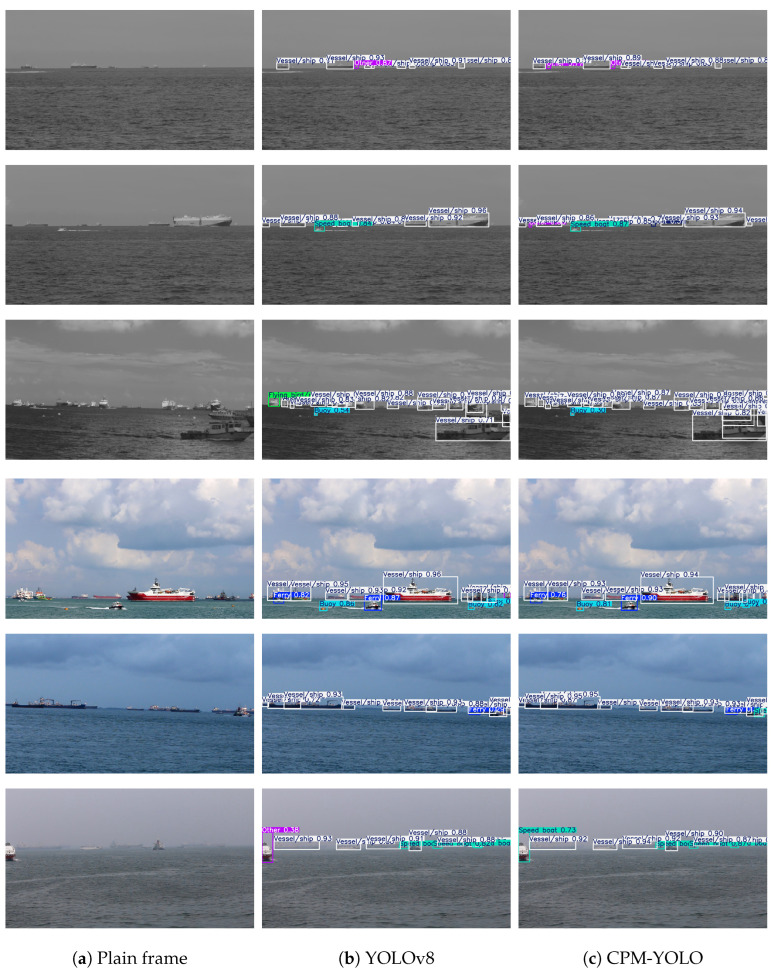
Detection results on the NIR (top) and VIS (bottom) datasets. The corresponding plain input frames are shown in (**a**), while the detection results of YOLOv8 and CPM-YOLO are shown in (**b**,**c**), respectively, to facilitate clearer visual comparison and target recognition.

To evaluate the detection performance of CPM-YOLO under different imaging conditions, Figure 10 presents qualitative comparisons on the NIR and VIS datasets. For each scene, the plain input frame is shown on the left for reference, while the detection results of YOLOv8 and CPM-YOLO are displayed in the middle and right columns, respectively. The selected samples cover scenarios with small targets, diverse categories, and complex backgrounds, highlighting the practical challenges of maritime detection.

In the NIR scenes, YOLOv8 exhibits missed detections and false alarms, particularly for small and distant vessels under low-contrast conditions. In contrast, CPM-YOLO demonstrates more reliable detection performance, achieving more accurate boundary localization and category classification, which indicates stronger robustness in low-light and long-range scenarios.

In the VIS scenes, YOLOv8 suffers from category confusion and target omissions in dense regions. Benefiting from enhanced multi-scale feature fusion and global attention modeling, CPM-YOLO is able to distinguish targets more clearly and maintain stable detection performance in crowded environments, especially for long-distance small targets and mixed multi-class scenes.

Overall, the visual results in Figure 10 confirm that CPM-YOLO consistently outperforms YOLOv8 in terms of detection accuracy, small-target recognition, and robustness in complex maritime scenes, providing a reliable foundation for subsequent multi-object tracking.

#### 4.4.4. Comparison of Vessel Tracking Methods

**Figure 11:**
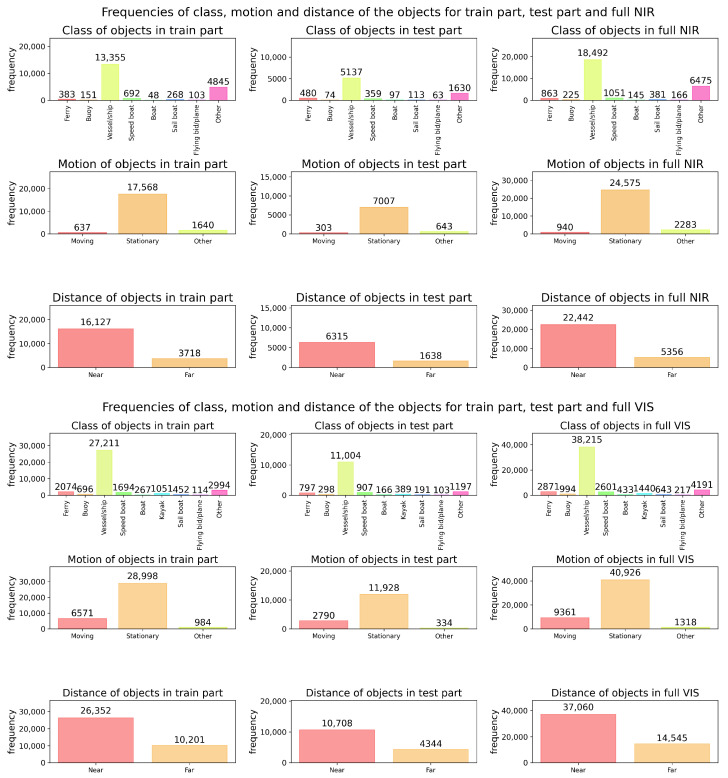
Statistical analysis of vessel categories, motion states, and distance distributions in the NIR (**top**) and VIS (**bottom**) datasets. For each dataset, the distributions are reported separately for the training set, test set, and the full dataset.

**Figure 12:**
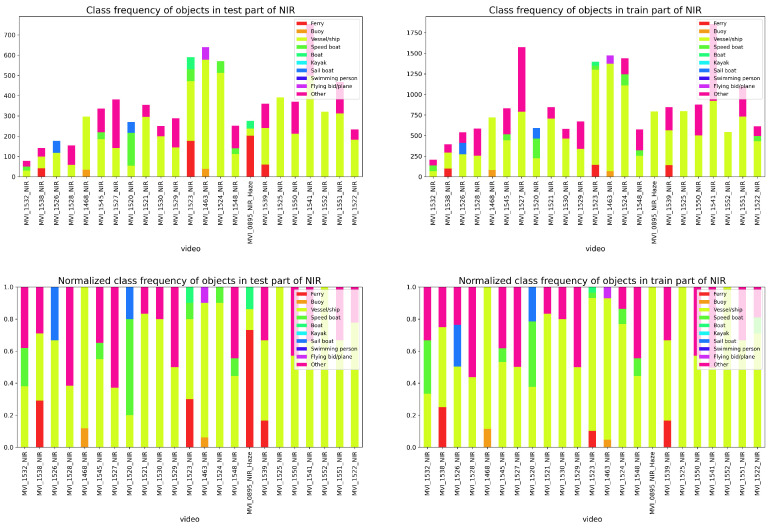
Frequency distribution of vessel categories in the test set (**left**) and training set (**right**) of the NIR dataset.

**Figure 13:**
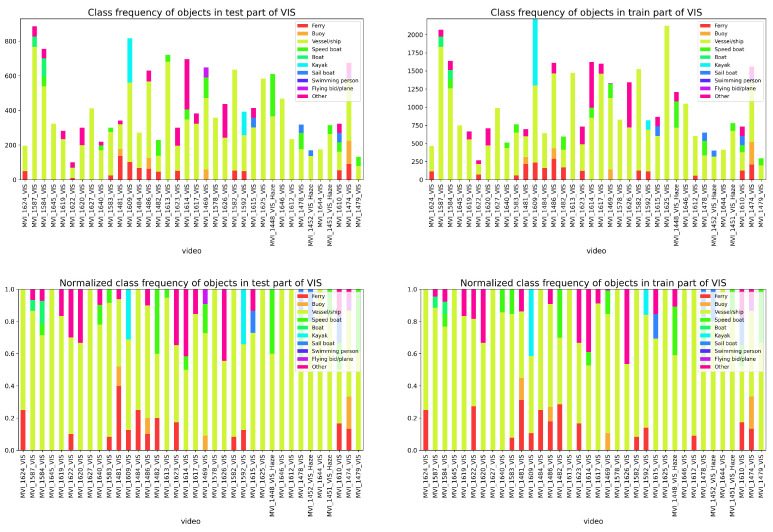
Frequency distribution of vessel categories in the test set (**left**) and training set (**right**) of the VIS dataset.

To systematically evaluate the performance of the proposed method on multi-vessel tracking, we combined CPM-YOLO with BOTSORT and compared it with leading tracking algorithms, including OCSORT, DeepOCSORT, and StrongSORT. For comprehensive assessment, we analyzed the NIR and VIS datasets: Figure 11 shows the overall distribution of vessel categories and motion states, while Figure 12 and Figure 13 present the frequency distribution of categories in the training and test sets for NIR and VIS datasets. Results indicate significant category imbalance and diversity in both datasets, with some classes underrepresented and others overrepresented, posing greater challenges for tracking robustness and cross-scene generalization.

**Figure 14:**
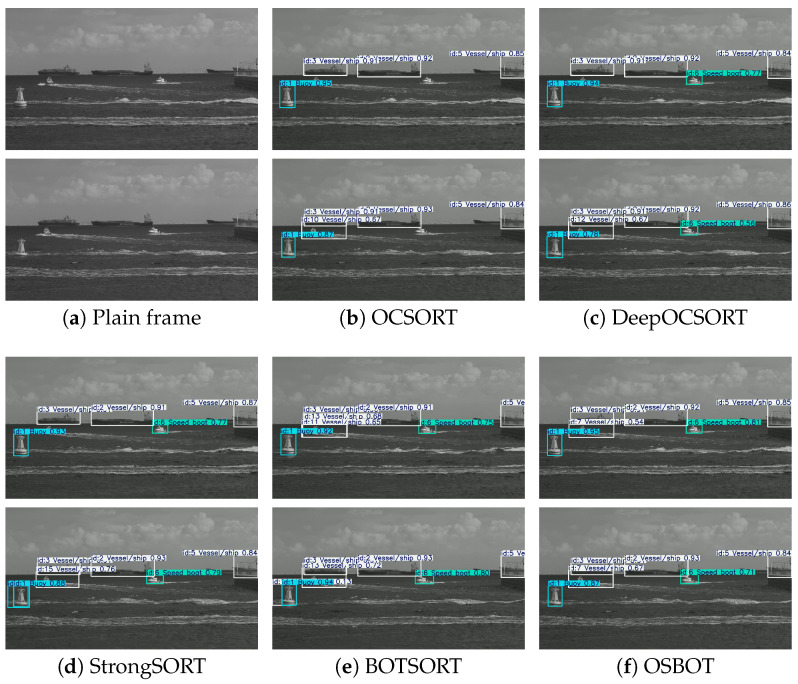
Comparison of vessel tracking results with different algorithms on the NIR dataset. For each scene, the plain input frame is shown in (**a**), followed by tracking results produced by OCSORT and DeepOCSORT in (**b**,**c**), respectively. The results of StrongSORT, BOTSORT, and OSBOT are shown in (**d**–**f**) for further comparison.

**Figure 15:**
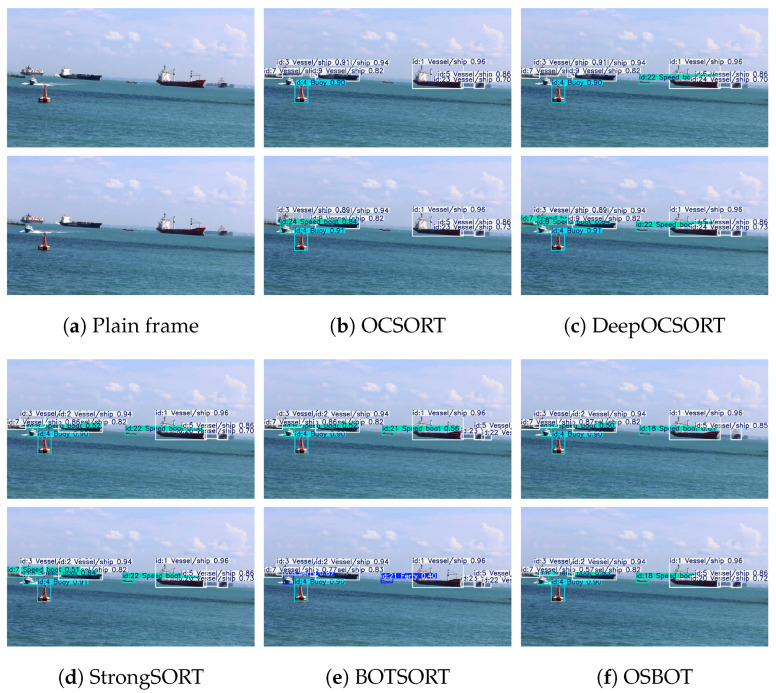
Comparison of vessel tracking results with different algorithms on the VIS dataset. For each scene, the plain input frame is shown in (**a**), followed by tracking results produced by OCSORT and DeepOCSORT in (**b**,**c**), respectively. The results of StrongSORT, BOTSORT, and OSBOT are shown in (**d**–**f**) for further comparison.

We further selected test video clips featuring small targets, dense vessels, heavy occlusion, and scale variation across frames to evaluate each tracking method in complex maritime scenarios. As shown in Figure 14 and Figure 15, CPM-YOLO with improved BOTSORT consistently outperforms OCSORT, DeepOCSORT, and StrongSORT in ID consistency, trajectory continuity, and occlusion recovery, showing a more stable association in diverse conditions. The consistent results on both NIR and VIS datasets demonstrate the cross-modal robustness and engineering potential of the proposed method.

From the experimental results, it can be observed that OCSORT tends to suffer from frequent trajectory interruptions in scenarios involving high-speed vessel motion. Moreover, under multi-target occlusion conditions, its observation-centric association mechanism struggles to maintain stable data associations, leading to pronounced ID losses. DeepOCSORT, by incorporating an adaptive appearance updating strategy, alleviates the impact of appearance variations caused by rapid target motion to some extent and thus exhibits improved stability in such scenarios. However, when occlusions persist for extended durations, the uncertainty of appearance information during occlusion still results in occasional tracking interruptions.

StrongSORT achieves competitive tracking performance when target appearance features remain relatively stable; nevertheless, its strong reliance on appearance cues makes it prone to ID conflicts in scenes involving stationary or slowly moving vessels, and its robustness to complex background variations and illumination changes remains limited. BOTSORT, by introducing geometric constraints and motion correction mechanisms, is capable of preserving trajectory continuity under occlusion. However, in cases where targets are spatially proximate or exhibit similar appearances, it may still erroneously fragment a single target into multiple trajectories.

In contrast, the proposed OSBOT (an enhanced ReID-based extension of BOTSORT) demonstrates superior overall performance in complex maritime scenarios characterized by cluttered backgrounds, frequent occlusions, and visually similar vessels. Benefiting from more discriminative and stable deep appearance feature modeling, OSBOT effectively suppresses trajectory duplication, maintains identity consistency and temporal continuity, and ultimately achieves more reliable multi-object vessel tracking.

## 5. Discussion

This study systematically addresses several critical challenges in multi-vessel detection and tracking within complex maritime environments, including small-target detection, large-scale variation, frequent occlusion, and severe background interference. The proposed CPM-YOLO framework comprises a multi-branch detection network that integrates lightweight feature representation with global attention modeling, providing a targeted solution to these issues. In addition, BOTSORT is incorporated and enhanced with re-identification mechanisms, yielding a high-precision and robust approach for vessel tracking.

Experimental results on both NIR and VIS datasets demonstrate that CPM-YOLO significantly outperforms the baseline YOLOv8 in terms of detection accuracy, recall, and mAP. Notably, CPM-YOLO achieves these improvements with lower parameter counts and a smaller model size, indicating an effective balance between performance and computational complexity. In MOT tasks, the improved BOTSORT algorithm demonstrates superior trajectory continuity, occlusion recovery, and identity consistency compared to methods such as OCSORT, DeepOCSORT, and StrongSORT, especially in challenging scenarios characterized by dense targets and noisy backgrounds.

The integration of the C2fCPCAGhost module, the P2 small-object detection branch, and the MHSA mechanism further enhances feature representation and enables more precise target perception. These advancements benefit not only the detection stage but also facilitate reliable association for downstream tracking tasks. As a result, the proposed system demonstrates substantial potential for intelligent maritime monitoring and channel safety management, effectively addressing real-world engineering challenges.

In comparison with previous studies, the proposed approach achieves a robust trade-off between accuracy and stability, offering new insights for the development of lightweight and efficient vessel detection and tracking systems. Nevertheless, certain limitations persist. For example, the method’s performance under extreme weather conditions or environments with very low visibility warrants further exploration. Moreover, the generalization ability of the system across various sensor modalities and diverse environmental scenarios remains an open question and should be investigated in future work.

Future research will focus on cross-modal information fusion, advanced spatiotemporal feature modeling, and the development of even lighter and more adaptable tracking modules. These research directions are expected to further enhance the robustness and applicability of multi-vessel detection and tracking methods across a wide range of scenarios and operational conditions.

## Figures and Tables

**Table 1:** Detection Model Comparison Results on the NIR Dataset.

Models	Precision	Recall	mAP@0.5	mAP@0.5:0.95	Parameters	Size/MB	FPS
YOLOv5	88.7%	74.4%	76.3%	49.4%	7,037,095	14.4	114
YOLOv7	91.2%	74.6%	74.9%	48.2%	6,308,131	12.3	127
YOLOv8	91.6%	71.2%	77.2%	52.1%	3,007,598	6.2	182
YOLOv11	93.7%	73.8%	77.8%	52.7%	2,591,490	5.6	191
YOLOv12	93.4%	71.8%	77.6%	53.8%	2,553,904	6.2	193
Ours	95.3%	76.1%	82.3%	57.5%	2,685,848	5.8	188

**Table 2:** Detection Model Comparison Results on the VIS Dataset.

Models	Precision	Recall	mAP@0.5	mAP@0.5:0.95	Parameters	Size/MB	FPS
YOLOv5	86.7%	76.4%	78.1%	49.5%	7,037,095	14.4	112
YOLOv7	90.7%	74.2%	75.3%	48.4%	6,308,131	12.3	126
YOLOv8	90.4%	72.1%	76.4%	50.6%	3,007,598	6.2	184
YOLOv11	93.2%	73.6%	76.8%	52.8%	2,591,490	5.6	192
YOLOv12	93.6%	72.2%	76.9%	53.6%	2,553,904	6.2	196
Ours	94.7%	76.8%	81.4%	56.8%	2,685,848	5.8	187

**Table 3:** Ablation Study Results of Detection Models on the NIR Dataset.

Baseline	C2fCPCAGhost	P2	MHSA	Precision	Recall	mAP@0.5	mAP@0.5:0.95	Parameters	Size/MB
✓				91.6%	71.2%	77.2%	52.1%	3,007,598	6.2
✓	✓			93.2%	71.8%	77.7%	53.6%	2,550,878	5.4
✓	✓	✓		94.2%	75.2%	81.8%	55.6%	2,447,512	5.3
✓	✓	✓	✓	95.3%	76.1%	82.3%	57.5%	2,685,848	5.8

**Table 4:** Ablation Study Results of Detection Models on the VIS Dataset.

Baseline	C2fCPCAGhost	P2	MHSA	Precision	Recall	mAP@0.5	mAP@0.5:0.95	Parameters	Size/MB
✓				90.4%	72.1%	76.4%	50.6%	3,007,598	6.2
✓	✓			92.3%	74.3%	78.3%	53.8%	2,550,878	5.4
✓	✓	✓		93.6%	76.2%	80.2%	55.5%	2,447,512	5.3
✓	✓	✓	✓	94.7%	76.8%	81.4%	56.8%	2,685,848	5.8

## Data Availability

The data presented in this study are available upon request from the corresponding authors. The data are not publicly available due to privacy/security considerations.
